# Mandibular overdentures retained by 1 or 2 implants: a 5-year randomized clinical trial on implant stability and peri-implant outcomes

**DOI:** 10.1007/s00784-024-05914-w

**Published:** 2024-09-16

**Authors:** Cláudio Rodrigues Leles, Gabriela Pereira de Resende, Nilva de Oliveira Martins, Lays Noleto Nascimento, Nadia Lago Costa, Murali Srinivasan, Martin Schimmel

**Affiliations:** 1 School of Dentistry, Federal University of Goias, Goiania, Brazil; 2 Clinic of General-, Special Care- and Geriatric Dentistry, Center of Dental Medicine, University of Zurich, Zurich, Switzerland; 3 Department of Reconstructive Dentistry and Gerodontology, School of Dental Medicine, University of Bern, Bern, Switzerland; 4 Division of Gerodontology and Removable Prosthodontics, University of Geneva, Geneva, Switzerland

**Keywords:** Edentulous mouth, Dental implant, Overdenture, Early dental implant loading, Implant stability quotient, Trials, Randomized clinical

## Abstract

**Aim:**

This is a report of the 5-year results of a two-group parallel randomized clinical trial comparing longitudinal implant stability, and clinical and radiographic peri-implant outcomes of mandibular overdentures retained by one (1-IOD group) or two (2-IOD group) implants.

**Methods:**

All participants received 4.1 mm diameter tissue-level implants (Straumann^®^ Standard Plus – SLActive^®^, Institut Straumann AG), installed in the mandible midline (1-IOD; *n* = 23) or the lateral incisor-canine area bilaterally (2-IOD; *n* = 24), and loaded after 3 weeks. Implant Stability Quotient (ISQ) was measured using a resonance frequency device (Osstell^®^ Mentor, Integration Diagnostics) at implant placement, after three weeks (loading), and at the 6-month, 1-, 3-, and 5-year follow-ups. Marginal bone loss and clinical implant outcomes (plaque, calculus, suppuration and bleeding) were assessed periodically up to 5 years after loading.

**Results:**

Only minor changes in marginal bone level were observed after 5 years (mean = 0.37; SD = 0.44 mm), and satisfactory and stable peri-implant parameters were observed throughout the 5-year follow-up. No significant differences between groups were found. Overall, the mean primary implant stability was considered high (> 70) for the two groups (1-IOD = 78.1 ± 4.5; 2-IOD = 78.0 ± 5.8). No noticeable changes were observed between implant insertion and loading. A marked increase was observed from insertion to the 6-month follow-up – the mean difference for the 1-IOD group was + 5.5 ± 5.5 (Effect size = 1.00), while for the 2-IOD group, the mean difference was + 6.0 ± 5.6 (Effect size = 1.08). No relevant changes were observed throughout the follow-up periods up to 5 years. Linear mixed-effect model regression showed no influence of the bone-related variables (*p* > 0.05) and the number of implants (*p* = 0.087), and a significant effect of the time variable (*p* < 0.001).

**Conclusion:**

Satisfactory peri-implant outcomes and stable secondary stability suggest good clinical performance and successful long-term osseointegration of the implants for single and two-implant mandibular overdentures. Using a single implant to retain a mandibular overdenture does not seem to result in detrimental implant loading over the five years of overdenture use.

**Clinical relevance:**

This study corroborates the use of a single implant to retain a mandibular denture.

## Introduction

Implant survival is a primary outcome for evaluating the efficacy and safety of implant treatments since it reflects the pivotal evidence of the success of the intervention at the implant level. However, implant failure is a highly undesirable outcome that is preceded by a series of negative events associated with the surrounding tissues, which can be identified in early stages and, when properly managed, may interrupt tissue derangement and prevent irreversible treatment failure. Therefore, when failure rates are expected to be low in implant clinical trials, surrogate endpoints, such as measures of peri-implant tissue health, low marginal bone loss and stability in implant stability parameters, emerge as valuable indicators of treatment success.

Healthy peri-implant tissues have become synonymous of implant success, and has been clinically determined by the absence of erythema, bleeding on probing, swelling and suppuration [1]. Primary stability is related to the mechanical engagement with cortical bone, while secondary stability is developed from the regeneration and remodeling of the bone and tissue around the implant after insertion, which is affected by the initial primary stability, bone formation, and remodeling [2]. Longitudinal (secondary) stability may be a sensitive and earlier measure of implant-related complications, allowing preventive interventions before more severe problems, such as implant loss, occur [3]. Therefore, the assessment of secondary stability offers a comprehensive and predictive approach to determining the long-term prognosis of implants both at placement and during function [4]. Low secondary stability may be indicative of overload and ongoing failure processes. Biomechanical overload can lead to bone damage and resorption and compromise osseointegration, decreasing implant stability.

Furthermore, it is known that in rehabilitating edentulous patients, the number of implants must be carefully planned to adequately support masticatory forces and avoid the risk of implant overload and, consequently, a decline in implant stability. International consensuses recommend a minimum of two implants for retaining a mandibular overdenture [5–7]. However, a simpler alternative using a single midline implant has been used with satisfactory results concerning patient-centered outcomes and implant survival. On the other hand, there is little information on the longitudinal stability when overdenture treatments with one or two implants are compared, especially in long-term follow-ups. Therefore, this study is part of a randomized clinical trial that aimed to compare the longitudinal changes in implant stability between overdentures retained by one or two implants. The condition of the peri-implant tissue health, considering radiographic and clinical parameters were also assessed. The study hypothesis is that the two treatments perform similarly concerning implant stability, marginal bone loss, and soft tissue health over five years of follow-up.

## Materials and methods

### Study design and sample

This is a report of the findings on the longitudinal implant stability as part of a two-group parallel randomized clinical trial comparing mandibular overdentures retained by a single (1-IOD group) or two implants (2-IOD group) opposing a conventional maxillary complete denture. This report was also produced following the CONSORT 2010 statement guidelines for randomized clinical reporting [8].

The complete study was carried out at the School of Dentistry of the Federal University of Goias, Brazil. The primary study protocol was registered at the ClinicalTrials.gov database (NCT03691285). The study followed the principles of the Declaration of Helsinki concerning the ethical principles for medical research involving human subjects, and the study protocol was approved by the research ethics committee of the Federal University of Goias (CAAE: 65240617.5.0000.5083). An amendment to the original protocol for the longitudinal 5-year follow-up was also approved (CAAE 5525322.4.0000.5083). All subjects involved in this clinical trial had provided their informed consent before inclusion in the study.

The detailed methodology for patient selection, randomization, and surgical and prosthetic procedures have been previously reported along with the 1-year results on patient-reported outcomes and masticatory function [9]. In summary, participants were edentulous patients wearing conventional dentures referred for mandibular overdenture treatment. Participants were assigned to the two groups using simple block randomization stratified by gender. A prior sample size calculation comprised 48 participants, 24 in each group.

### Intervention

All study participants received a new set of complete dentures, and after a minimum 3-month period, they were scheduled for implant treatment. All implant surgeries were planned, including a radiographic assessment of bone availability and edentulous ridge morphology to confirm sufficient bone height in the midline regions and bilateral canine areas for placement of one or two regular-diameter implants with a length of 8, 10, or 12 mm. All participants received 4.1 mm diameter tissue-level implants (Straumann^®^ Standard Plus – SLActive^®^, Institut Straumann AG, Basel, Switzerland), installed according to the manufacturer’s recommendations, using a flapped surgery and early loading protocol. The position of the implants varied according to the treatment group – for the 1-IOD group, a single implant was inserted in the mandible midline. For the 2-IOD group, two implants were inserted bilaterally in the lateral incisor-canine area. The final insertion torque was checked with a torque wrench set at 25 Ncm. A healing abutment was connected to the implants, the tissues were sutured, and the mandibular denture was relieved and relined with a temporary soft relining material (Soft Comfort, Dencril, São Paulo, Brazil).

Implant loading was performed after 21 days. A 3.4 mm retentive titanium spherical anchor abutment (Straumann 048.439, Institute Straumann AG, Basel, Switzerland) was connected at 35 Ncm with a torque wrench. The corresponding elliptical matrix (Straumann 048.456, Institute Straumann AG, Basel, Switzerland) was incorporated into the mandibular denture using a chairside technique intraoral pickup with self-curing acrylic resin, and the dentures firmly occluded. The excess material was trimmed, and the patient was instructed about the proper use of the overdenture, oral hygiene measures, and the need for regular maintenance.

### Outcomes

A set of relevant outcomes were assessed in this study and reported elsewhere, including surgical morbidity and complications [10, 11], life impact benefits and functional improvement [9], and incidence of prosthodontic complications [12]. This report focuses on the longitudinal assessments of the peri-implant tissue health, and the changes in implant stability, which refers to the primary/mechanical and secondary/biological implant stability.

Bleeding on probing was considered a sign of gingival inflammation, and was valued as present or absent. Additionally, a Gingival Index was used to score as follow: 0 – no bleeding when the probe is passed along the gingival margin; 1 – isolated bleeding, spots present; 2 – blood forms a confluent red line on margins; 3 – heavy or profuse bleeding.

Plaque and calculus accumulation around the implant were assessed with the naked eye and with a periodontal probe around the attachment. The plaque/calculus index was scored as: 0 – no detection of plaque; 1 – plaque only recognized by running probe across the smooth marginal surface of implant; 2 – plaque seen by naked eye; 3 – abundance of soft matter/calculus.

Marginal bone loss was assessed by measuring the peri-implant marginal bone level on standardized digital periapical radiographs obtained in the follow-up visits. The peri-implant bone level was measured using the Cliniview™ software (Cliniview™, Instrumentarium Company). The vertical distance (in millimeters) from implant platform to the first bone-to-implant contact was measured at the mesial and the distal aspects of the implant. Changes in bone level were calculated by subtracting the marginal bone levels values from baseline measurements (3-month level).

Implant stability measures were performed quantitatively using a resonance frequency assessment device (Osstell^®^ Mentor, Integration Diagnostics, Gothenburg, Sweden) and the corresponding SmartPeg transducer (Type 04 – Article no.100350). The resonance frequency value was automatically converted into an Implant Stability Quotient (ISQ) value, ranging from 1 to 100, where higher values represent greater implant stability. High stability is achieved with ISQ values ≥ 70 ISQ, medium stability between 60 and 69, and low stability with values < 60 [13]. Three measurements were taken on the buccal, right, and left sides of the transducer, and each reading was performed in triplicate. A mean value was calculated to provide the ISQ value for the individual implant.

Six time points for ISQ measurements were planned: immediately after implant placement; 21 days after surgery – implant loading; at the 6-month follow-up of overdenture use; and the 1-year, 3-year, and 5-year follow-ups.

#### Independent variables

In addition to the number of implants and time after implant insertion, other potential predictors were also assessed as influential variables, including patient’s age and sex, and clinical features such as the classification of the residual ridge (high well-rounded, knife-edge, flat, depressed) [14], level of ridge resorption (normal vs. severe), the prosthodontic prognostic classification scores (Prosthodontic Diagnostic Criteria) according to the American College of Prosthodontists for edentulous patients [15], ridge height (in millimeters), final implant insertion torque (in Newtons. cm), and bone type classification according to the Lekholm & Zarb classification [16].

### Data analysis

Descriptive statistics was used to summarize the data on baseline characteristics and the repeated clinical, radiographic and ISQ measurements. In case of statistically significant differences, the effect size for within-subjects (repeated measures) design was calculated by dividing the mean difference by the standard deviation of the difference, and the interpretation of the effect size values was: ≤0.2 is a small effect, near 0.5 is a medium effect, and ≥ 0.8 is a large effect.

Multivariate analysis using Generalized Estimating Equations (GEE) was used to assess the effect of time treatment groups for the peri-implant variables. Linear Mixed-effect Model (LMM) regression was performed to model the variation in ISQ over time. Both GEE and LMM regression were used due to the dependent repeated measures for the longitudinal data (within-implant measures) and the occurrence of multiple implants within an individual patient in the 2-IOD group. In addition, the regression models tested the influence of the time-dependent changes in ISQ as influenced by the treatment group, final insertion torque, and bone site features (bone type, anatomic features). The final model estimates were expressed as regression coefficients and their standard errors. The IBM-SPSS 24.0 software was used for data analysis, and statistical significance was set at *p* < 0.05.

## Results

A total of 47 patients were included in this study to received implant treatment, 23 in the 1-IOD group and 24 in the 2-IOD group, 35 (74.5%) females, and ages ranged from 44 to 81 years (mean = 65.4; SD = 8.5) the time of implant surgery. Hence, 71 implants were placed and assessed in this study. The main characteristics of the implant sites are depicted in Table 1.

**Table 1:** Characteristics of the implant sites, according to the treatment groups. Data are expressed as frequencies (and percentages

		1-IOD (*n*-23)	2-IOD (*n* = 24)	Total (*n* = 71)
Ridge form	Well-rounded	6 (26.1)	26 (54.2)	32 (45.1)
	Flat	9 (39.1)	16 (33.3)	25 (35.2)
	Regular	6 (26.1)	6 (8.3)	10 (14.1)
	Knife-edge	2 (8.7)	2 (4.2)	2 (5.6)
Ridge resorption	Normal	14 (60.9)	28 (58.3)	42 (59.2)
	Severe	9 (39.1)	20 (41.7)	29 (40.8)
PDI index*	Class II	2 (8.7)	0 (0.0)	2 (4.3)
	Class III	18 (78.3)	22 (91.7)	40 (85.1)
	Class IV	3 (13.0)	2 (8.3)	5 (10.6)
Alveolar height	≤ 27 mm	12 (52.2)	26 (54.2)	38 (53.5)
	≥ 28 mm	11 (47.8)	22 (45.8)	33 (46.5)
Bone type	Type I	5 (21.7)	4 (8.7)	9 (13.0)
	Type II	12 (52.2)	14 (30.4)	26 (37.7)
	Type III	5 (21.7)	22 (47.8)	27 (39.1)
	Type IV	1 (4.3)	6 (13.0)	7 (10.1)

* Prosthodontic Diagnostic Index – measured at the patient level

**Fig. 1:**
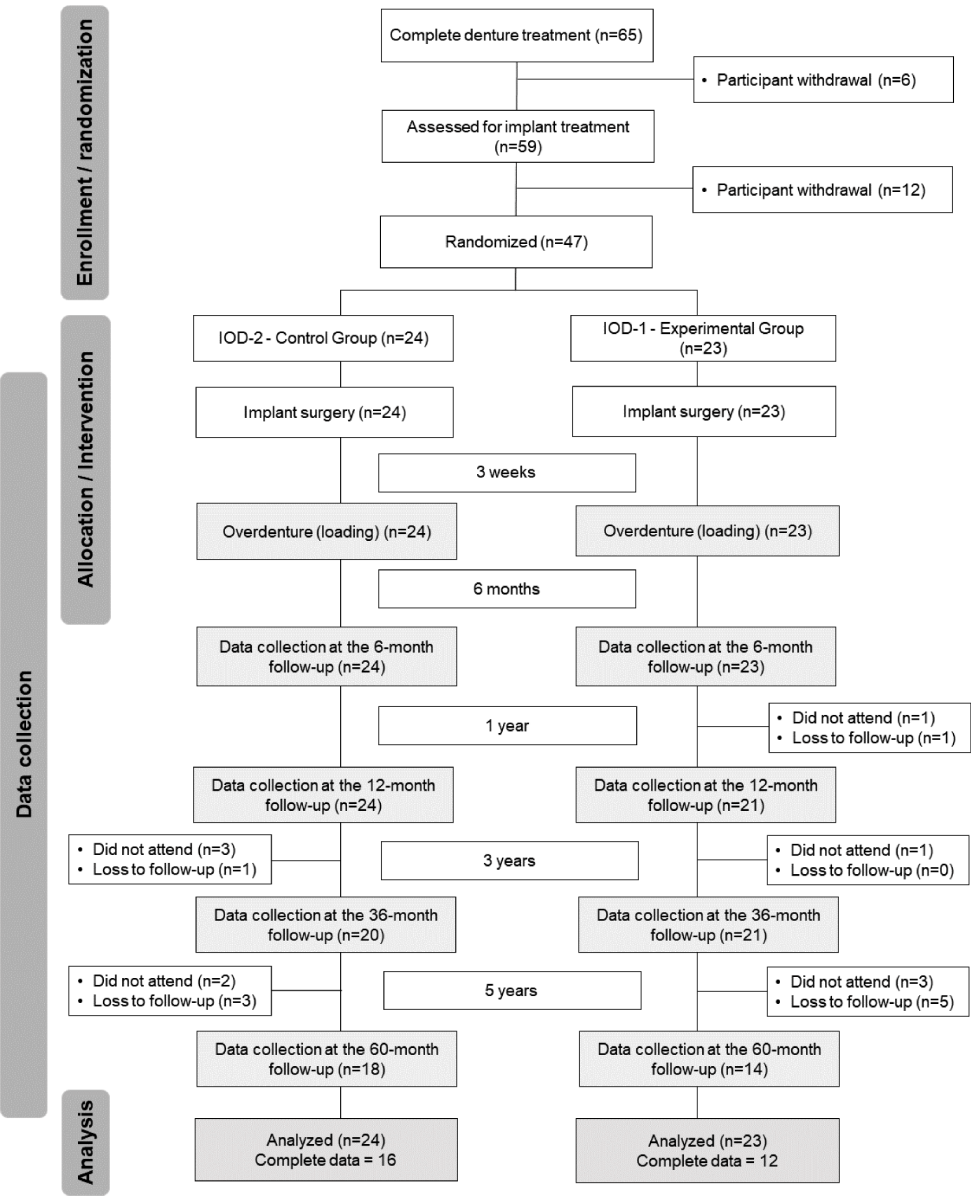
Study flowchart

The complete study flowchart with the number of patients included in each follow-up visit is detailed in Fig. 1. No implant failure occurred after implant loading and overdenture use. From the sample of patients who received implant treatment in the 1-IOD group (*n* = 23), there was one deceased patient at the 1-year follow-up and one who didn’t attend the follow-up visit. At the last follow-up, 14 patients were assessed in the 1-IOD group and 18 in the 2-IOD group. The numbers of patients with complete data collection were 12 and 16 in the 1- and 2-IOD groups, respectively.

**Fig. 2:**
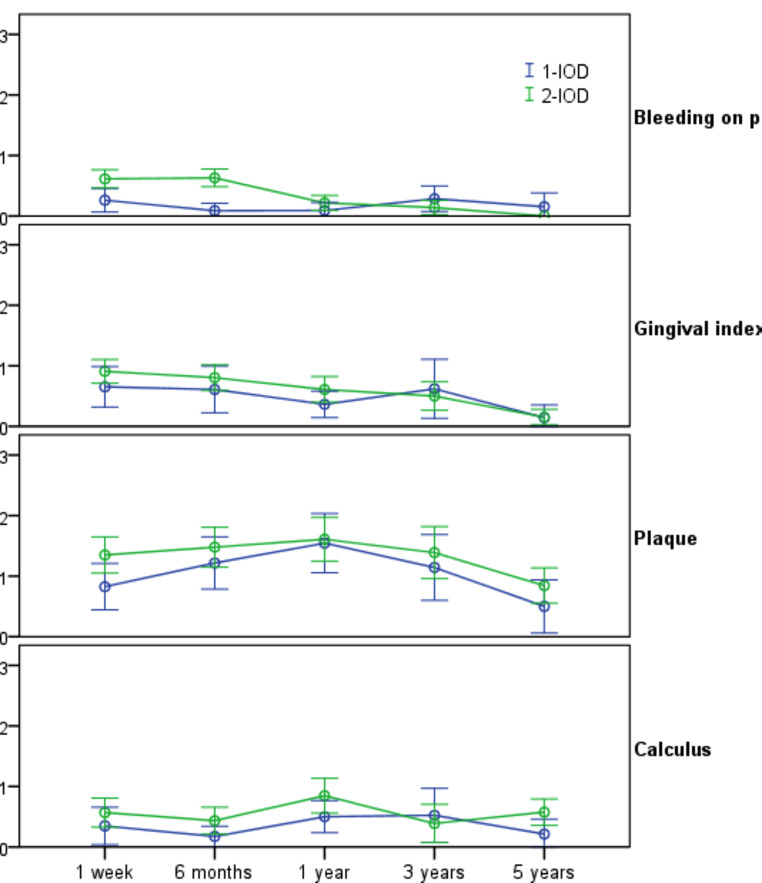
Changes in peri-implant outcomes according to the treatment groups and study time points

Figure 2 depicts the variation in peri-implant soft tissue parameters. Bleeding on probing was observe in only 25.9% of the measurements, and only 10.1% showed gingival index ≥ 2. No significant changes were observed throughout the longitudinal assessments. The presence of suppuration was not noted and was not presented in the graph. GEE regression models revealed a significant reduction in the risk of bleeding on probing (OR = 0.49; 95%CI = 0.24–0.99; *p* = 0.045) and on gingival index (OR = 0.09; 95%CI = 0.003–0.26; *p* < 0.001) in the 5-year follow-up. The magnitude of the reductions after 5 years were considered large for bleeding on probing (ES = 0.84) and the gingival index (ES = 0.81), and a small effect on plaque index was observed (ES = 0.32). No significant effect of time was observed for the other peri-implant parameters. Similarly, no significant effect of the treatment group (1- vs. 2-IOD) was detected.

The overall marginal bone loss after 5 years ranged from 0 to 1.49 mm (mean = 0.37 ± 0.44) and 86.0% of the implants were lower than 1.0 mm at the 5-year follow-up. When treatment groups are compared, the mean marginal bone loss values were 0.39 ± 0.39 mm and 0.36 ± 0.46 mm for the 1-IOD and 2-IOD groups, respectively. The between-group difference was not statistically significant (mean difference = 0.04 mm; *p* = 0.793).

**Fig. 3:**
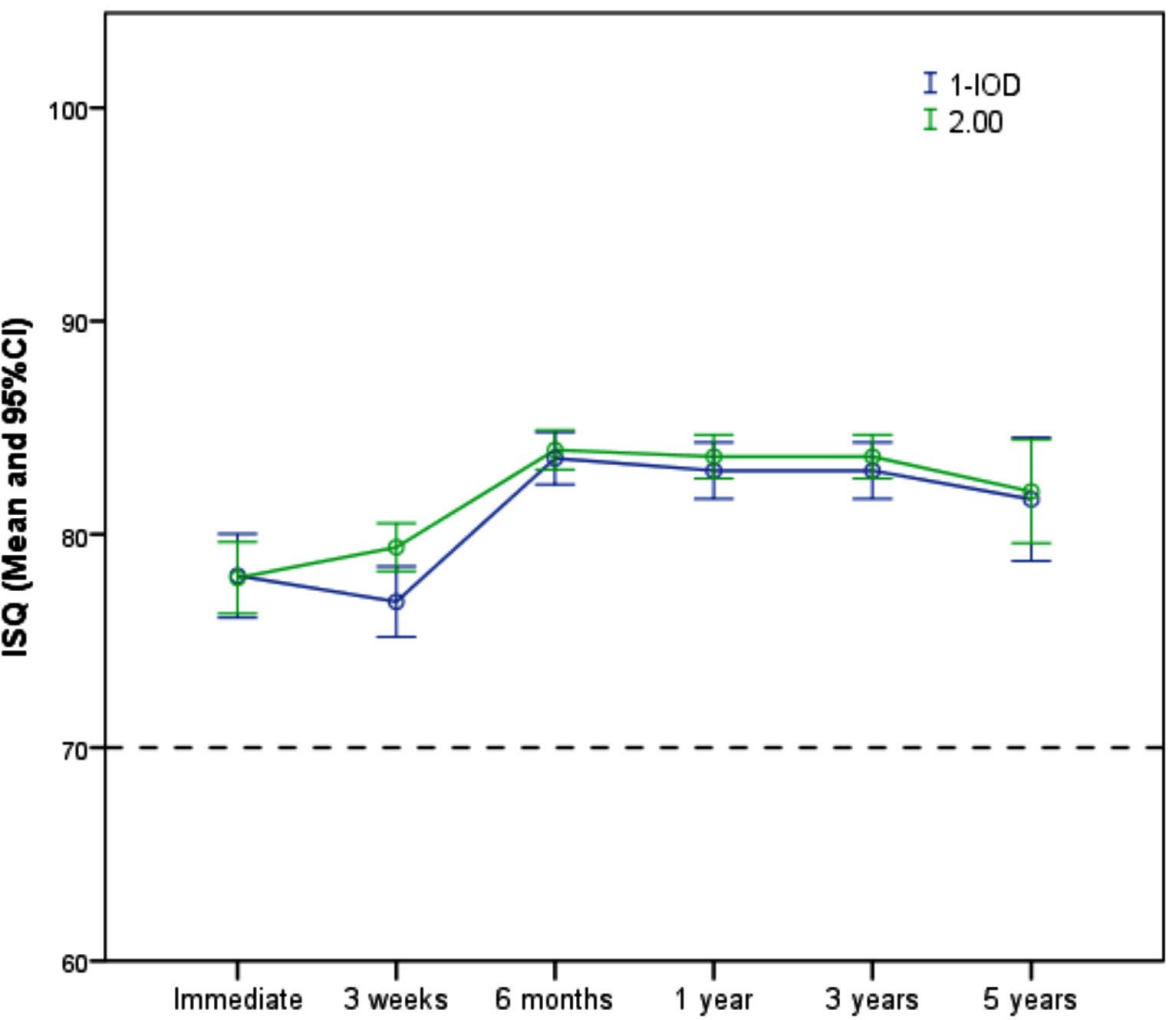
Changes in the Implant Stability Quotient (ISQ) values according to the treatment groups and study time points

Figure 3 shows the variation in ISQ values at each study’s time point, according to the treatment group. The mean primary implant stability was considered high (> 70) for the two groups (1-IOD = 78.1 ± 4.5; 2-IOD = 78.0 ± 5.8). Considering all data, 385 valid measurements were obtained, 375 (97.4%) were considered high (≥ 70), 8 (2.1%) were medium, and only 2 (0.5%) were low, with no difference between groups (*p* = 0.117). The mean final implant insertion torque was similar for the 1-IOD (22.6 ± 6.5 Ncm) and 2-IOD (22.7 ± 7.4) groups.

No noticeable changes were observed between implant insertion and loading (21 days) for the two groups. Then, a marked increase was observed from insertion to the 6-month follow-up. In this period, the mean ISQ of the 1-IOD group changed from 78.1 ± 4.5 to 83.6 ± 2.8 (mean difference = + 5.5 ± 5.5; Effect size = 1.00), while for the 2-IOD group, the mean ISQ changed from 78.0 ± 5.8 to 84.0 ± 3.2 (mean difference = + 6.0 ± 5.6; Effect size = 1.08). No relevant changes were observed throughout the follow-up periods after the 6-month follow-up.

Regression analysis revealed no influence of the bone-related variables (*p* > 0.05). The parameters of the final LMM regression model are detailed in Table 2. There was no effect of the number of implants on the overall ISQ values (*p* = 0.087) and a significant effect of the time variable (*p* < 0.001). ISQ values were stable from surgery until the loading time three weeks after surgery (*p* = 0.530), followed by a significant improvement at the 6-month follow-up (*p* < 0.001), which remained stable until the 5-year follow-up.

**Table 2:** Regression parameter estimates

Parameter		Estimate	Std. Error	*p*-value
Intercept		78.16	0.65	< 0.001
Treatment group	1-IOD	-0.666	0.388	0.087
	2-IOD	Reference		
Time points	5 years	3.91	1.10	0.001
	3 years	5.43	0.75	< 0.001
	1 year	5,45	0.75	< 0.001
	6 months	5.84	0.73	< 0.001
	3 weeks (loading)	0.49	0.79	0.530
	Immediate (surgery)	Reference		

Dependent Variable: ISQ

## Discussion

This study corroborates findings from previous studies that showed comparable outcomes following treatment with mandibular overdentures retained by one or two implants. Specifically, this study showed the overall peri-implant conditions were satisfactory and tended to present a slight improvement after 5 years. The measures of primary stability were high at the time of the early loading protocol after three weeks, and the secondary stability increased after one year and remained stable for up to 5 years. No differences were found in comparing the treatments with one or two implants; therefore, the study hypothesis was confirmed.

Findings revealed that none of the cases presented signs of poor soft or hard tissue health, and implants did not achieved the clinical criteria of peri-implant mucositis, and peri-implantitis [17], independently from the number of implants for overdenture retention. Proper oral hygiene, including regular and thorough cleaning of the overdenture attachments, is essential to prevent peri-implant disease [18]. Moreover, anatomic and individual factors may play important role for maintenance of peri-implant health. Low levels of keratinized mucosa are associated with the accumulation of plaque [19]. The good longitudinal performance of the tissue-level implants in our study reinforces their use as a long-term safe and reliable alternative to the available portfolio of dental implants, which is particularly suitable for elderly patients [20].

Concerning the implant stability measures, in both groups, ISQ values remained consistently above 75, as indicated by the literature as parameters for implants considered highly stable [2, 21], and, therefore, successful osseointegration was achieved. This explains the 100% survival rate of implants after loading with the overdenture and may suggest that the load transferred to the implant by the fixation system and the functioning of the prosthesis are within an acceptable biomechanical level, even when there is only a single implant to retain the overdenture. Furthermore, secondary stability is maintained in the medium and long term, establishing it as a viable and safe treatment since biomechanical overload can lead to bone damage and resorption and compromise osseointegration, decreasing implant stability.

Likely, the 1-IOD and 2-IOD do not differ significantly in implant stability values due to several factors. First, both treatment modalities were designed to provide adequate stability and support for the mandibular overdenture, resulting in comparable findings. Furthermore, clinical and implant-level aspects that can directly influence ISQ changes include surgical technique, design, and implant surface [22].

During the 3-week healing period, ISQ remained high and stable until the early loading stage. This pattern is consistent with the gradual increase in stability since surgery, as observed by Hicklin et al. [23], who showed that a functional occlusal loading of implants with a hydrophilic, moderately rough endosseous surface three weeks after placement appears to be a safe and predictable treatment option in healed sites in the posterior mandible [23]. Another factor that may explain the increase or stability in ISQ values after surgery is the relatively low insertion torques, which are associated with the promotion of rapid secondary stability, as by admitting less osseocompression and tension. Thus, a smaller implant-bone contact area presumably allows the rapid formation of new bone in the vicinity of the implant without significant resorption of pre-existing bone tissue [24, 25]. As a result, there may not have been a decrease in ISQ that could be measured after 21 days.

One limitation of this longitudinal trial was the failure to assess all study participants after the 3-year follow-up. One-third of the included patients didn’t conclude the longer-term clinical assessments, which is attributed to the medium/long-term nature of the evaluation. This attrition rate could limit the study power and may influence the interpretation of the findings and generalization of these results, as observed in other long-term studies on single-implant overdentures [26, 27]. However, loss to follow-up rates were similar in the two groups and may not be a relevant issue to conclude that the two treatment groups were comparable concerning longitudinal implant stability. Therefore, a systematic statistical bias is not assumed when comparing these two groups. In similar studies, as evidenced in our work, loss to follow-up was also notable.

This study provides additional clinical evidence on the feasibility of the single-implant overdenture concept. However, other factors related to the patient’s biological characteristics and the implant may influence treatment success. Therefore, future studies should include peri-implant outcomes and the quality of supporting tissues, which may play a predictive role in the prognosis of a single-implant retained overdenture treatment.

## Conclusions

Findings of this longitudinal study showed that the mandibular overdenture treatment with one or two implants performed similarly, resulting in satisfactory peri-implant soft tissue health and low levels of marginal bone loss over the 5-year follow-up period. ISQ values were stable from the 6-month until the 5-year follow-up, and no differences were observed between the implants retaining a single and two-implant mandibular overdenture. The stable secondary stability suggests successful long-term osseointegration of the implants for the two treatments.

Therefore, this study showed that the two treatments were similar regarding implant-related outcomes over the five years of overdenture use. This reinforces the concept that using a single implant to retain a mandibular overdenture can be a reliable alternative to the standard treatment with two implants.

## Data Availability

No datasets were generated or analysed during the current study.

## Declarations

### Trial registration

ClinicalTrials. Gov ID: NCT03691285 – Mandibular Overdenture Retained by 1 or 2 Implants: A Cost-effectiveness Analysis (IOD1-2). Study start: 09/04/2017
